# Interpretable unsupervised learning enables accurate clustering with high-throughput imaging flow cytometry

**DOI:** 10.1038/s41598-023-46782-w

**Published:** 2023-11-23

**Authors:** Zunming Zhang, Xinyu Chen, Rui Tang, Yuxuan Zhu, Han Guo, Yunjia Qu, Pengtao Xie, Ian Y. Lian, Yingxiao Wang, Yu-Hwa Lo

**Affiliations:** 1 Department of Electrical and Computer Engineering, University of California, San Diego, La Jolla, CA 92093 USA; 2 NanoCellect Biomedical, Inc., San Diego, CA 92121 USA; 3 Department of Bioengineering, Institute of Engineering in Medicine, University of California, San Diego, 9500 Gilman Drive, La Jolla, CA 92093-0435 USA; 4 Department of Biology, Lamar University, Beaumont, TX 77710 USA

**Keywords:** Functional clustering, High-throughput screening, Machine learning

## Abstract

A primary challenge of high-throughput imaging flow cytometry (IFC) is to analyze the vast amount of imaging data, especially in applications where ground truth labels are unavailable or hard to obtain. We present an unsupervised deep embedding algorithm, the Deep Convolutional Autoencoder-based Clustering (DCAEC) model, to cluster label-free IFC images without any prior knowledge of input labels. The DCAEC model first encodes the input images into the latent representations and then clusters based on the latent representations. Using the DCAEC model, we achieve a balanced accuracy of 91.9% for human white blood cell (WBC) clustering and 97.9% for WBC/leukemia clustering using the 3D IFC images and 3D DCAEC model. Above all, although no human recognizable features can separate the clusters of cells with protein localization, we demonstrate the fused DCAEC model can achieve a cluster balanced accuracy of 85.3% from the label-free 2D transmission and 3D side scattering images. To reveal how the neural network recognizes features beyond human ability, we use the gradient-weighted class activation mapping method to discover the cluster-specific visual patterns automatically. Evaluation results show that the automatically identified salient image regions have strong cluster-specific visual patterns for different clusters, which we believe is a stride for the interpretable neural network for cell analysis with high-throughput IFCs.

## Introduction

Imaging flow cytometry (IFC), combining the high throughput and multiparameter capabilities of traditional flow cytometry with morphological and spatial contents from imaging, has gained increasing interest in broad fields of medicine, drug discovery, immunology^1^, cell biology, microbiology, marine biology^2^, etc. A major challenge associated with high-throughput imaging flow cytometry is the enormous amount of imaging data generated in each experiment. Human-interpretable morphological features such as texture, granularity, and size-related features are usually extracted as a low-dimensional representation of images from which researchers perform the gating analysis for cell classification. However, these hand-crafted sets of image features are far from a thorough representation of the biological properties of cells. Furthermore, biological insight could be present in the high-dimensional feature space not recognizable by human vision, especially when the features are convoluted and not intuitively obvious.

With the advances in deep learning and artificial intelligence (AI), neural networks have been applied to recognize features embedded in cell images to aid cell classification from both labeled and label-free imaging data. Most cell classification neural networks demonstrated to date use training sets consisting of specific cell types for supervised learning. Recent works demonstrated impressive performance by combining high-throughput imaging techniques with the IFC systems and data-driven machine learning algorithms for label-free cell classifications. Chen et al.^3^ applied the artificial neural network to a time-stretch quantitative phase imaging system and achieved a balanced accuracy of 96.4% in the classification of cancerous cells. Wu et al.^4^ combined the frequency-shifted optofluidic time-stretch quantitative phase imaging (OTS-QPI) technique and a convolutional neural network (CNN) autoencoder to demonstrate a balanced accuracy of 96% in the classification of leukemia from healthy WBCs. Rui et al.^5^ used a three-dimensional (3D) imaging flow cytometer system and a customized UNet model to achieve a balanced accuracy of 92.3% for human WBC type classification. Despite the promising results, all these approaches are based on supervised machine learning, which relies on the ground truth labels of the training dataset. However, in many biomedical applications such as drug discovery^6–8^, cell-type discovery^9,10^, biomarker discovery^11,12^, and T-cell receptor (TCR) identification for immunotherapy^13,14^, to name a few, it is time-consuming, cost-forbidden, or simply unfeasible to obtain the ground truth labels of sufficient quality or quantity required for supervised machine learning.

To address the above limitations, we need techniques to cluster image data generated by the IFC without any prior knowledge of input labels, an approach known as unsupervised machine learning. Since the performance of traditional clustering algorithms is compromised as the dimensionality of the input dataset increases, we introduce a novel unsupervised deep convolutional autoencoder-based clustering (DCAEC) model that can learn from a high-level representation. DCAEC is an extension of deep autoencoders (DAEs)^15,16^ and deep convolutional autoencoders (DCAEs)^17^, which can learn the latent representations of the input dataset through an unsupervised approach. These models contain an encoder and a decoder, having the encoder project the raw input (i.e., 3D cell image) into the latent representations and the decoder reconstructs the latent representations back to the original data (i.e. an AI-constructed 3D cell image).

Our approach is divided into two steps, the training step and the clustering step. In the training step, the deep convolutional autoencoder model is utilized to obtain good representations from the raw input images by minimizing the image reconstruction error. In the clustering step, the decoder of the DCAE model is suppressed and the encoder part is transferred to the DCAEC model followed by the Gaussian Mixture Clustering Model (GMM) to cluster the latent representations. We have explored two DCAEC models, 3D DCAEC, and fused DCAEC based on the types of raw input images. The 3D DCAEC model takes 3D side-scattering (SSC) cell images from a 3D IFC system as inputs and the fused DCAEC model takes both the 3D SSC cell images and two-dimensional (2D) transmission cell images as inputs from the same system. In all cases, unlabeled cell images are used for training and testing and the fluorescent labels of the cells are only used to evaluate the clustering performance at the end.

Three experiments were conducted to evaluate the DCAEC model performance with unsupervised learning and label-free cells. For the white blood cell (WBC) clustering experiment, we demonstrated a balanced accuracy of 91.9% for three-part clustering. In the blood cancer detection experiment, the leukemia cells were successfully clustered from the healthy WBCs with a balanced accuracy of 97.9% and specifically 100% accuracy in predicting leukemia. We further examined the model performance from a protein translocation experiment where proteins are localized in the nucleus or cytoplasm in different cells. Here the fused DCAEC model was deployed and a balanced accuracy of 85.3% was achieved. In contrast, no feature extraction algorithms based on human recognizable features such as size, shape, granularity, contrast, entropy, etc. can detect protein translocation in cells without fluorescent protein labeling, indicating that the neural network with unsupervised learning can recognize hidden features in high-dimensional feature space beyond human capabilities.

The system’s high performance, especially its “superhuman” capabilities, motivates the study of explainable AI to understand how AI discovers cluster-specific visual patterns and uses them to distinguish one cluster from another. We first learn a deep neural network $N$ to identify the correlation relationship between pixels in single-cell images and cluster labels. Then for each image $i$, we leverage the learned network $N$ and the Gradient-weighted Class Activation Mapping (Grad-CAM) method^18^ to faithfully localize salient image regions that are most relevant to the cluster label $c$ of this image. The localized regions are utilized to interpret why image $i$ is assigned to cluster $c$. Grad-CAM computes the gradient information flowing into the last convolutional layer of the deep network $N$ to obtain a localization map and understand the relevance of individual pixels in an input image to its cluster label. This study is performed on the protein translocation images. Results show that the automatically identified salient image regions have strong cluster-specific visual patterns and can clearly distinguish different clusters. Such cluster-tailored visual patterns are difficult to be identified by human experts manually.

## Results

### Cell clustering workflow using deep unsupervised learning

**Figure 1:**
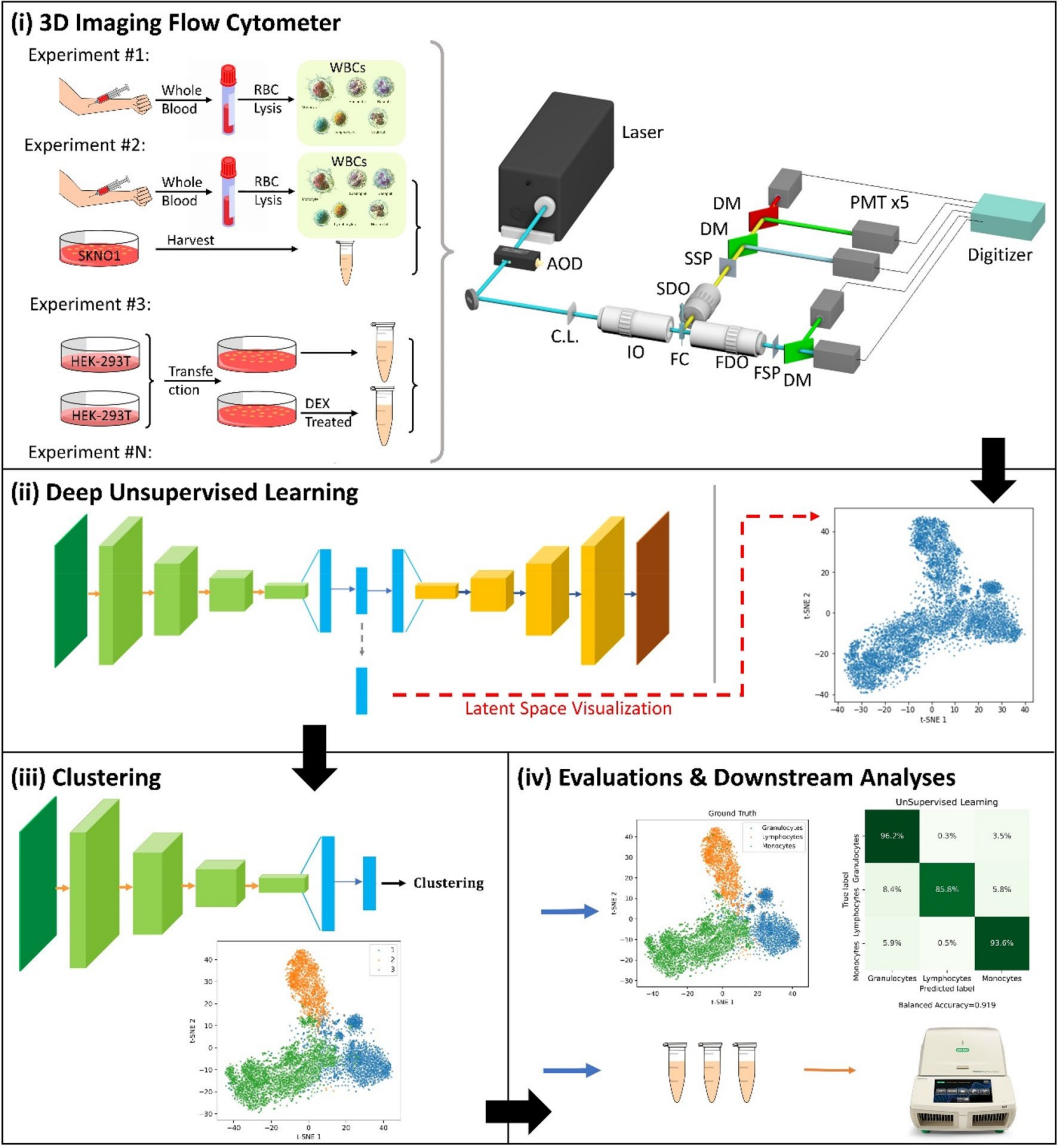
Deep unsupervised learning-based single-cell clustering workflow. (**i**) After the sample preparation, cells are examined using the 3D-IFC system. (**ii**) The deep unsupervised learning model takes cell images as inputs and is trained to encode the inputs into latent representations. t-SNE visualization of the trained results of latent space representations. (**iii**) Model-predicted labels were generated at the clustering step. t-SNE visualization of the clustering result. (**iv**) For post-evaluation, the model-generated labels are compared with the ground truth labels, and the confusion matrix is produced. qRT-PCR can also be used for downstream analysis after the clustering step. AOD, acousto-optic deflector; CL, cylindrical lens; IO, 20 × /0.42 illumination objective; SDO, 10 × /0.28 side detection objective; SSP, side spatial filter; DMs, dichroic mirrors; FDO, forward detection objective; FSP, forward spatial filter; PMT, photomultiplier tube.

Figure 1 shows the overall workflow of deep unsupervised learning-based cell clustering. After the sample preparation, cells are premixed and examined by the 3D-IFC. Suspended cells are hydrodynamically focused in the flow cell cuvette, forming a single cell stream with a concentration of ~ 500 samples/uL. Cells are illuminated by a scanning light sheet (at 200 kHz scanning rate) when they pass through the laser interrogation area^5,19–21^. The 2D transmission image signal and the 3D side-scattering image signal of each cell are detected and processed in parallel^20,21^. The transmitted light passes through a spatial slit filter and is detected by a photomultiplier tube (PMT), and the 2D cell transmission image is constructed by a 2D temporal-spatial transformation algorithm^22^. Simultaneously, the side scattering signal (90-degree from the scanning incident beam) passes through a side spatial filter with a series of pinholes and is detected by a PMT, and the 3D cell SSC image is constructed by a 3D temporal-spatial transformation algorithm^22^. The preprocessed 2D transmission images and 3D SSC images become the inputs of the customized deep convolutional autoencoder (DCAE). The DCAE is trained in an end-to-end manner to encode high-dimensional data input images into a low-dimensional latent space. At the clustering step, the Gaussian Mixture Model (GMM) clustering method is embedded in the deep convolutional autoencoder-based clustering model (DCAEC) to cluster the encoded low-dimensional data. For evaluation purposes, the clustering results are compared with the ground truth labels. The confusion matrix shows the accuracy of the deep unsupervised learning-based cell clustering approach.

### Application to human white blood cells clustering

White blood cells (WBCs) are generated in the bone marrow and lymphoid tissues and are an essential part of the immune system. Here we use unsupervised WBC clustering to study the feasibility of our unsupervised learning method and, importantly, to set the reference for WBC distribution of healthy samples in contrast to leukemia cells that are difficult to obtain with controlled properties as ground truth samples.

The WBC clustering experiment is divided into two phases, the training phase and the clustering phase. During the training phase, the DCAE is trained using the dataset obtained from the Veri-Cells Leukocyte Kit prepared from lyophilized human peripheral blood leukocytes^5^. In the clustering phase, the DCAE pre-trained weights are transferred to the DCAEC model and WBCs from the whole blood of healthy donors (San Diego Blood Bank) are clustered.

**Figure 2:**
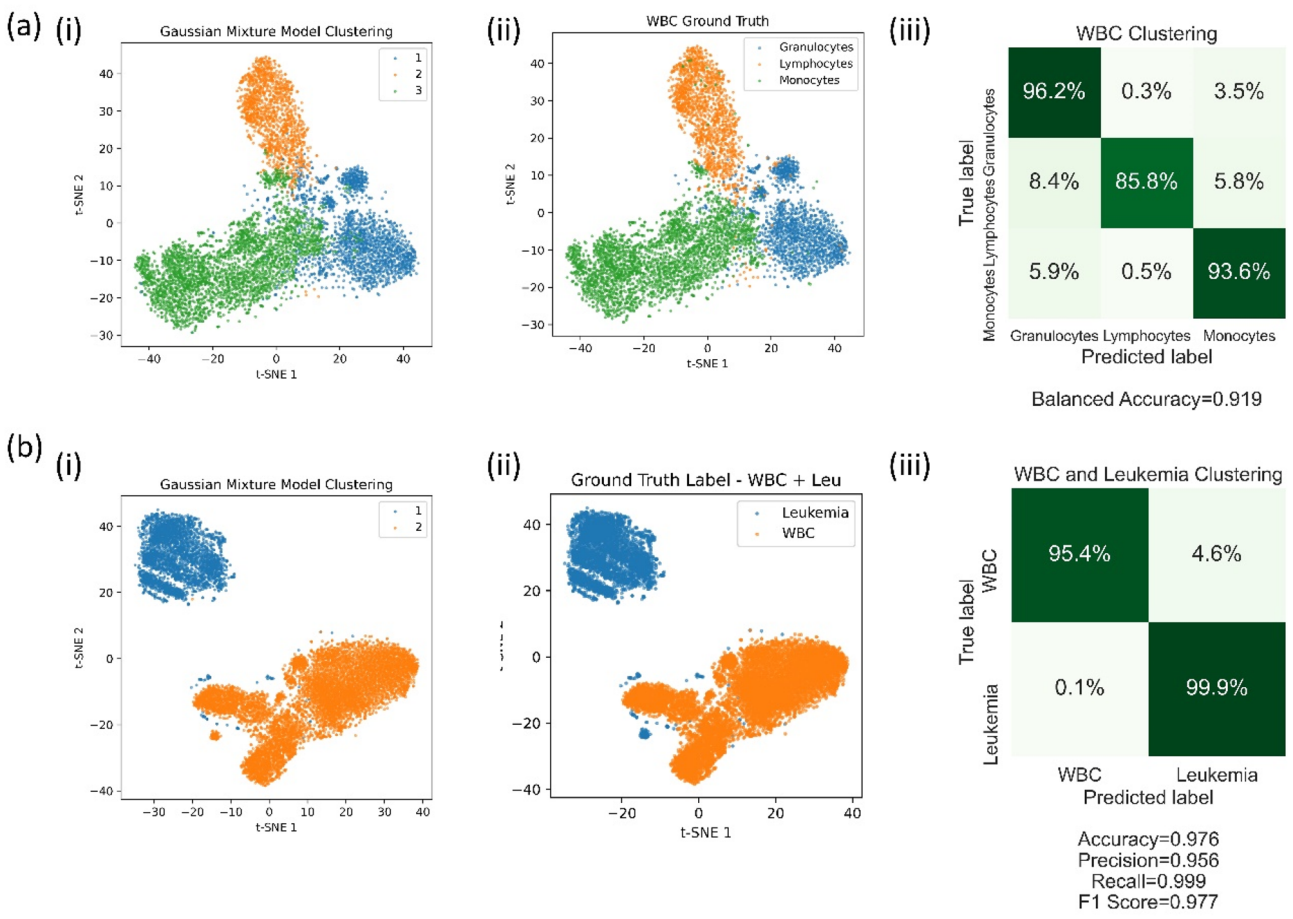
Results of (**a**) white blood cells (WBCs) clustering experiment and (**b**) blood cancer detection experiment. (i) Clustering predicted labels from the DCAEC model. (ii) Ground truth labels. (ii) Corresponding confusion matrix.

The WBCs clustering results are shown in Fig. 2a-i. For the post-evaluation of the clustering accuracy, we compare the clustering results with the ground truth labels from antibody staining (Fig. 2A-ii). The confusion matrix in Fig. 2A-iii shows that a balanced accuracy of 92% is achieved.

### Detection of leukemia using unsupervised learning

Blood cancer is among the most common cancer types in the United States^23^ and acute myeloid leukemia (AML) is one type of blood cancer characterized by infiltration of the bone marrow by proliferative, clonal, and abnormally differentiated cells from hematopoietic stem cells^24^. Traditional AML diagnostic procedures use morphological assessments of bone marrow specimens/blood smears or antibody-specific blood tests^24^. A quick and reliable method for label-free leukemia clustering using an imaging flow cytometer (IFC) and deep learning can potentially simplify the diagnosis process and aid early detection. In the following, we demonstrate that the DCAEC model can correctly cluster leukemia from healthy WBCs using unsupervised learning and 3D SSC images from an imaging flow cytometer system.

In a proof-of-concept experiment, the patient-derived SKNO1 AML cells are spiked into the white blood cells of healthy donors (obtained from SDBB). We bypass the training step by directly transferring the pre-trained weights of the DCAE model from the Veri-Cells dataset to the DCAEC model. At the clustering step, the model encodes the input 3D SSC images into a low-dimensional latent space and creates clusters based on the encoded latent space variables. Figure 2B shows the clustering results of a mixture of SKNO1 cells and healthy white blood cells and the corresponding confusion matrix. The results show that the DCAEC model can successfully cluster leukemia cells out of healthy WBCs at a confidence level approaching 100%.

### Detection of protein translocation using unsupervised learning

The signaling and particle transport in eukaryotic cells are usually associated with protein translocation which is essential for correct cell functions^25,26^. Abnormal protein localization is an indication of many human diseases. For example, Breast Cancer Gene 1 (BRCA1) protein is normally localized in the cell nucleus to perform the functions of DNA repair and cell-cycle checkpoints. However, in cancer cells, p53 promotes the BRCA1 nuclear export causing the re-localization of BRCA1 in the cytoplasm^26–28^. Traditional wet lab experiments for protein translocation detection in human cancer tissues rely heavily on protein-specific antibody labeling, a process that can be expensive, laborious, and time-consuming^29,30^. In the following, we combine the unlabeled cell images from an IFC and deep unsupervised learning to demonstrate a label-free method to cluster the enhanced green fluorescent protein (GFP)- glucocorticoid receptor (GR) translocated and un-translocated HEK-293 T cells from a mixture of both.

**Figure 3:**
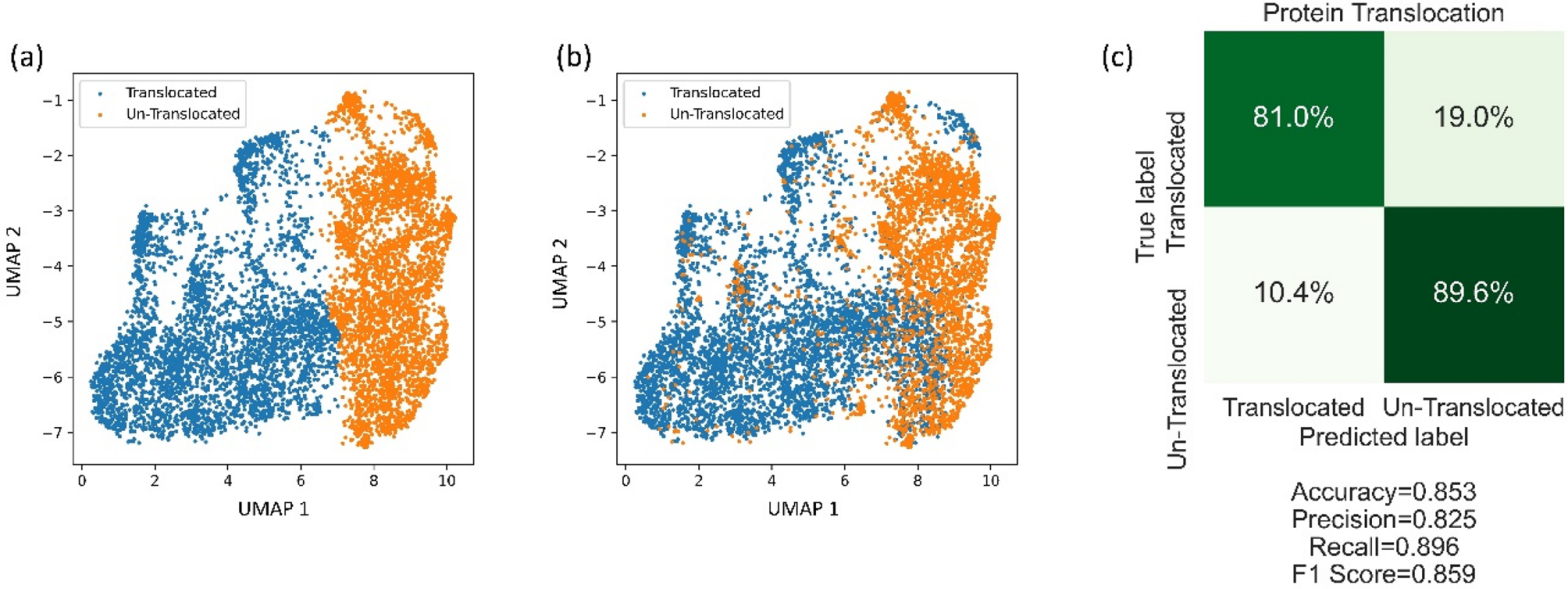
Clustering results of protein translocation experiment. (**a**) Clustering predicted labels from the DCAEC model. (**b**) Ground truth labels. (**c**) Corresponding confusion matrix.

Two dishes of HEK-293 T cells are cultured at 37 °C with 5% CO_2_. Cells are transfected by the same amount of pEGFP-GR plasmid when they reach 70% confluency. One dish of cells is stimulated by GR agonists (here we use dexamethasone)^31^. The majority of EGFP-GR is localized in the cytoplasm in unstimulated cells while dexamethasone induces translocation of EGFP-GR into the nucleus. The cells are imaged by an imaging IFC that produces both 3D SSC images and 2D transmission images simultaneously. At the training step, the fused DCAE model takes both 2D transmission and 3D SSC images as inputs and is trained to encode them into low-dimensional latent space. The GMM is embedded to cluster the low-dimensional pattern variables at the clustering step. The clustering results are shown in Fig. 3. We achieved a balanced accuracy of 85.3% by using these label-free images with the unsupervised learning method.

The high classification accuracy of protein-translocated cells without labeling is surprising for several reasons. First, the cell images have neither the resolution nor the contrast to distinguish the distribution of a particular type of protein. Secondly, all attempts to use human-recognizable features such as cell size, cell shape, image contrast, granularity, etc., or any combination of these image features to detect protein translation have failed (Supplementary Fig. 3). Therefore, questions arise about how AI can produce superior cell classification results than humans and whether there exist any non-intuitive features in the high-dimensional feature space not recognizable by human vision. If so, could these AI-detectable features be new biomarkers or contain new biological insight or phenotypical characteristics? Although we are not able to provide unequivocal answers to these profound and important questions, in the following we discuss our investigation of interpretable AI for cell classification by their images, as the first step of finding how AI recognizes cell features that escape human vision.

### Automatically discover cluster-specific visual patterns for protein translocation images

Given the clusters identified from single-cell images by AI, we are interested in finding what visual patterns AI uses to separate one cluster from another. To investigate this problem, we first train a deep neural network $N$ to discover the correlation between image pixels and cluster labels. Then we leverage $N$ and the Gradient-weighted Class Activation Mapping (Grad-CAM) method^18^ to identify salient regions in each image that are most relevant to the cluster label of this image. Finally, we analyze these automatically identified salient regions of images in the same cluster to check whether they show common visual patterns and check whether these visual patterns can distinguish different clusters.

**Figure 4:**
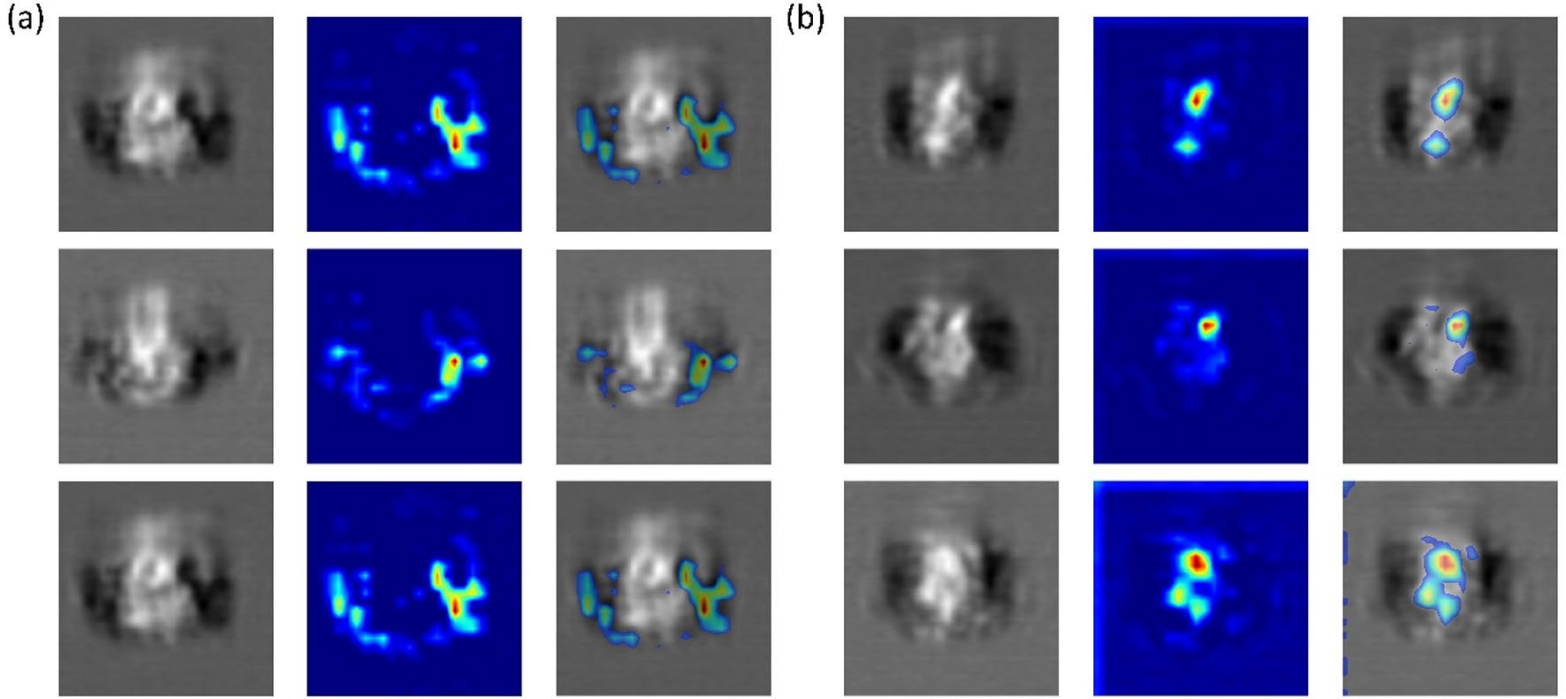
Grad-CAM visualizations of the last conv-layer of the encoder. (**a**) Protein translocated group. (**b**) Protein Un-Translocated group. For each group, the first column is the input images; the second column is the generated heatmap; the third column is the overlay results of the first two columns.

We perform this study on protein translocation images since humans fail to identify any visual patterns that can distinguish cells with and without protein translocation from unlabeled cell images. We first extract visual representations of these images using the pre-unsupervised-trained 2D DCAE model. On top of these representations, we learn a deep neural network $N$ to classify which cluster each image belongs to. Training details and results of $N$ are deferred to the supplementary material. For each image, we first use the trained $N$ to calculate the classification logits. Then this image, the predicted logits, and network weights of $N$ are fed into Grad-CAM to localize regions in the image that are most relevant to the logits. Figure 4 shows localization heatmaps (warmer color denotes higher relevance) for randomly selected images from the two clusters. As can be seen, heat maps of images in the same cluster are visually similar. This explains why these images are grouped into the same cluster. In addition, heat maps of protein-translocated images are visually quite different from those of protein un-translocated images, which shows that it is possible to discover visual patterns that clearly distinguish different clusters.

We quantitatively check whether localized salient regions can distinguish different clusters. For each heatmap, we threshold it into a binary mask (white color denotes larger relevance). Detailed signal processing workflow can be found in Supplementary Fig. 4. We split these masks into five folds.

For each fold $f$, we train a simple CNN-based classifier (model architecture can be found in Supplementary Fig. 5) on the other four folds to predict the mask’s cluster labels and evaluate the trained classifier on $f$. Table 1 shows the fivefold cross-validation results. As can be seen, the classification performance is very high, which demonstrates that the two clusters of masks can be easily distinguished. (More detailed evaluation results can be found in Supplementary Fig. 6 and Supplementary Table 1).

**Table 1:** fivefold cross-validation results for the classification of binary masks.

Fold/metrics	Accuracy	Precision	Recall	F1	AUC
Fold 1	0.959	0.962	0.960	0.961	0.985
Fold 2	0.957	0.962	0.958	0.960	0.988
Fold 3	0.960	0.968	0.960	0.963	0.990
Fold 4	0.959	0.964	0.960	0.961	0.988
Fold 5	0.962	0.962	0.962	0.962	0.987

## Discussion

In summary, we have demonstrated a deep convolutional autoencoder-based clustering model for accurate clustering of cell images produced by high-throughput imaging flow cytometry. The DCAEC is an unsupervised deep embedding algorithm that first encodes the input images into the latent representations and the clustering is based on the latent representations. Comprehensive evaluations show that the DCAEC model offers an accurate clustering with a decent clustering accuracy. Two DCAEC models are examined based on the input data formats. A balanced accuracy of 91.9% and 97.9% are achieved in the WBC clustering experiment and blood cancer detection experiment using the 3D DCAEC model. The fused DCAEC model is developed for a more difficult task, protein translocation clustering, and a balanced accuracy of 85.3% is achieved. Attempting to understand how the CNN produces results unattainable by human-recognizable image features, we apply the technique of gradient-weighted class activation mapping (Grad-CAM) to reveal how the neural network classifies cells and forms clusters from cell images. For the challenging case of protein translocation detection, the Grad-CAM results reveal insight how AI captures features in the cell image that escape human vision.

The results of our study suggest that the DCAEC model, together with imaging flow cytometers that can capture 2D and/or 3D images of individual cells without the interference of neighboring cells offer unique capabilities for cell classification and cell type discovery, especially in conditions where a sufficient quantity of high-quality, reliable ground truth samples are unobtainable due to practical or cost reasons.

In the current implementation, the DCAE model is trained in an end-to-end manner. It can be modified for more advanced training procedures (e.g., incorporating the greedy layer-wise training method^32^). The encoder part is directly transferred to the DCAEC model followed by a GMM model to make a unified clustering model. Our current approach takes advantage of a deep neural network to encode the input images into the latent representations followed by clustering analysis. However, the current approach treats feature space learning and clustering as two separate procedures and the overall performance is not optimized. A natural next step would be to embed a clustering objective into the DCAE framework, which will likely yield more accurate and compact latent representations^33–35^. Finally, while we have shown the interpretation results based on the 2D transmission images to make the results friendly for human visualization, a similar approach can be applied to 3D SSC images as well.

## Materials and methods

### Sample preparation and image acquisition

#### White blood cell clustering experiment

White blood cells were obtained from whole blood after lysing the red blood cells (RBCs). Whole blood from healthy donors was purchased from San Diego Blood Bank (SDBB) for research purposes. The detailed RBC lyse protocol is described in the supplementary materials. The protocol for the dataset from the Veri-Cells Leukocytes can be found in our previous publication^5^. For post-evaluation purposes, the WBC sample was immuno-stained with anti-CD14 and anti-CD66b antibodies for phenotyping, establishing the ground truth labels for three WBC types—monocytes, granulocytes, and lymphocytes. The detailed staining protocol is discussed in the supplementary materials. After flowing the WBCs through the 3D-IFC system, 3D SSC images of each cell were collected and stored as a 3D image stack with 80 × 80 × 80 pixels^3^, corresponding to a field of view of 20 × 20 × 20 μm^3^.

#### Blood cancer clustering experiment

Normal WBCs were collected under the same method as the WBC clustering experiment but from a different healthy donor. The SKNO1 leukemia cell line was cultured and fixed prior to the experiment. For post-evaluation purposes, the Leukemia cells were fluorescently stained with the carboxyfluorescein succinimidyl ester (CFSE) Cell Proliferation Kit (Ex/Em 492/517 nm, Cat. 34554, ThermoFisher). The detailed culturing and staining protocol are listed in the supplementary materials. The mixture of leukemia cells and normal WBCs were examined by the 3D-IFC, and the 3D SSC images were collected.

#### Protein translocation experiment

**Figure 5:**
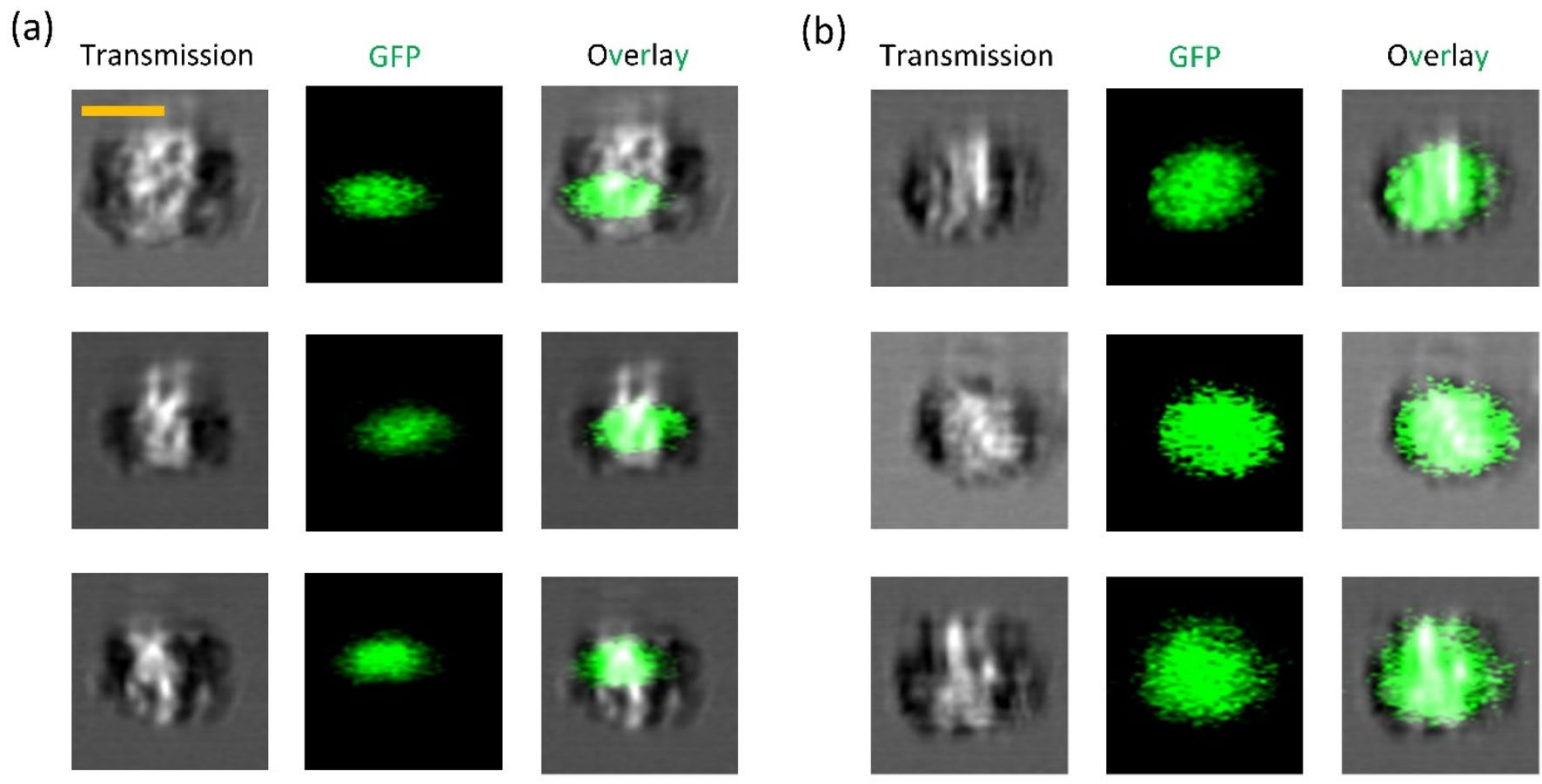
Exemplary images of protein translocation experiment. (**a**) Protein translocated group. (**b**) Protein un-translocated group. Transmission, green fluorescence, and overlay images generated from 3D-IFC. Scale bar: 10 μm.

The pEGFP-GR (Plasmid #47504, Addgene) DNA was extracted from bacteria after 18 h of culture using the Zyppy miniprep kit (#D4020). HEK293T cells were transfected with pEGFP-GR DNA using Lipofectamine 3000 (Catalog #L3000001, ThermoFisher Scientific) when they reached ~ 70% confluency. After stable expression of the EGFP-GR for 18–24 h after transfection, one dish of HEK-293 T cells was treated with dexamethasone (Catalog #tlrl-dex, Invitrogen). Both treated and untreated cells were harvested and fixed using 4% Paraformaldehyde prior to imaging. The dual-modality 3D-IFC can produce both 2D transmission images and 3D SSC images. In this experiment, 2D transmission and 3D SSC images were simultaneously collected for each cell. The 2D transmission images have 80 × 80 pixels^2^ for a field of view of 20 × 20* μ*m^2^. The ground truth labels for post-evaluation were obtained from the area ratio between the GR-GFP protein distribution represented by the 2D fluorescent area and the total cell area from the 2D transmission image. Exemplary images of translocated and un-translocated groups are shown in Fig. 5.

### Deep unsupervised learning—deep convolutional autoencoder-based clustering

Traditional deep autoencoder (DAE) is generally composed of two stacked fully connected layers, one as an encoder and the other as a decoder^36^. Unlike a DAE model, the deep convolutional autoencoder (DCAE) incorporates the convolutional and deconvolutional layers as part of the encoder and decoder instead of only fully connected layers^37^ to leverage the advantages of a convolutional neural network for image processing, especially for 3D image processing^38^. Sparse interactions, parameter sharing, and equivariant representations are key distinguishable properties of CNN in translation latent features^39^.

In this paper, two DCAE models are developed, the 3D DCAE model and the fused DCAE model. The 3D DCAE model takes the input of a 3D side-scattering image and 3D convolutional operations are implemented in the convolutional and deconvolutional layers. In the encoding part, several convolutional layers are trained to encode the input images into latent representations followed by a fully connected layer to translate the learned 3D features to a linear n-dimensional latent space. In the decoding part, a fully connected layer followed by several deconvolutional layers is trained to convert the latent representations back to the original input images. In the training step, the DCAE model is trained in an end-to-end manner to optimize the averaged mini-batch reconstruction error (mean-square-error, MSE) between the input image and the reconstructed image (Eq. 1). The mini-batch averaged MSE loss $L_{MSE}$ can be expressed as

1 $$L_{MSE} = \frac{1}{N}\mathop \sum \limits_{j = 1}^{N} \frac{1}{{M_{3d} }}\mathop \sum \limits_{i = 1}^{{M_{3d} }} \left( {x_{3d,i,j} - \hat{x}_{3d,i,j} } \right)^{2}$$

where $x_{3d}$ and $\hat{x}_{3d}$ are the input 3D SSC image and model-generated image, respectively, $M_{3d}$ is the flattened SSC image vector dimension, $N$ is the batch size in the mini-batch.

In the clustering step, the decoding part of the DCAE model is inactivated, and the pre-trained weights of the encoder are transferred to the deep convolutional autoencoder-based clustering (DCAEC) model. After encoding the input images into a latent space, a Gaussian Mixture clustering model (GMM) with k-means initialization^40^ is embedded to perform the clustering.

The fused DCAE model combines the 3D DCAE and the 2D DCAE, one taking 3D SSC images as inputs and the other taking 2D transmission images as inputs. Image features from both 3D SSC images and 2D transmission images are encoded into the latent spaces through two DCAE models. In the training step, the fused DCAE model is trained to optimize a weighted loss that consists of the mini-batch averaged MSE loss from the two DCAE models (Eq. 2).

2 $$L_{MSE} = w_{1} L_{MSE,2d} + \left( {1 - w_{1} } \right)L_{MSE,3d} = \frac{1}{N}\mathop \sum \limits_{j = 1}^{N} \left( {\frac{{w_{1} }}{{M_{2d} }}\mathop \sum \limits_{i = 1}^{{M_{2d} }} \left( {x_{2d,i,j} - \hat{x}_{2d,i,j} } \right)^{2} + \frac{{1 - w_{1} }}{{M_{3d} }}\mathop \sum \limits_{i = 1}^{{M_{3d} }} \left( {x_{3d,i,j} - \hat{x}_{3d,i,j} } \right)^{2} } \right)$$

where $x_{2d}$ and $\hat{x}_{2d}$ are the input 2D transmission image and model-generated image, respectively, $M_{2d}$ is the flattened transmission image vector dimension, $w_{1}$ is the mean-square-error loss weight assigned to the two image modalities.

**Figure 6:**
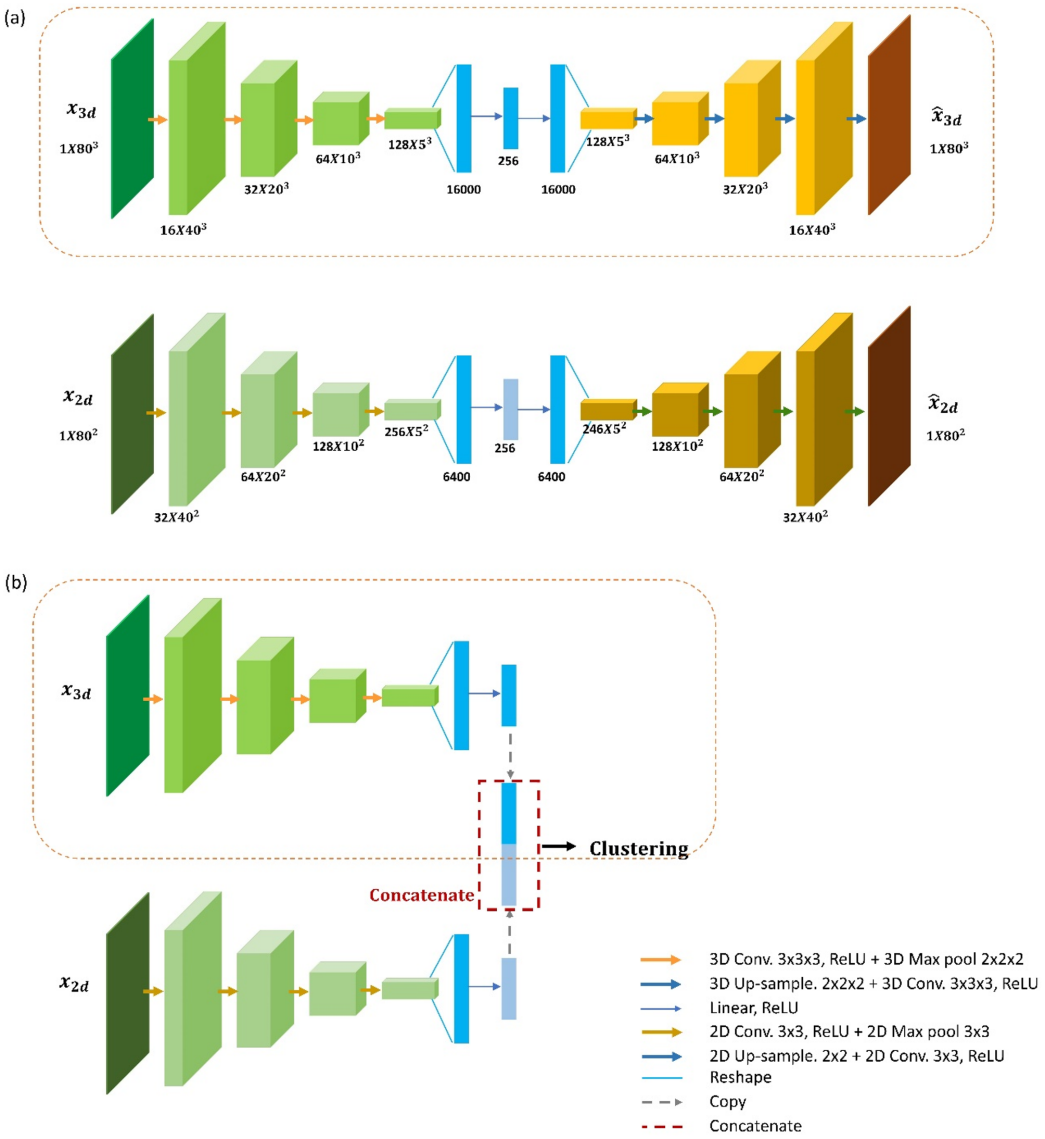
DCAEC model architecture. (**a**) Unsupervised training step, the DCAE model was trained in an end-to-end manner to encode the inputs to latent space. (**b**) Clustering step, the decoder part is suppressed and the clustering model uses the latent representations to form clusters. The orange rectangular shows the 3D DCAEC model which takes the 3D SSC cell images as input. Each box corresponds to a multi-channel feature map. The number of channels and feature size are denoted at the bottom of the box. Different types of arrows denote different operations according to the legends.

In the clustering step, the fused DCAEC model consists of the encoders from the fused DCAE model. The latent spaces are concatenated together with a GMM model to cluster the concatenated latent spaces. Figure 6 shows the model architecture of DCAEC.

Although the model is trained in an unsupervised manner to perform the clustering, the ground truth labels for each experiment are obtained for post-evaluation purposes. A confusion matrix is produced for each experiment with clustering predicted labels on the x-axis and truth labels on the y-axis. The balanced classification precision, recall, and ${F}_{1}$ score are reported for the blood cancer clustering experiment and protein translocation experiment. Detailed definitions of the above reports can be found in the supplementary materials. The balanced accuracy is calculated from the arithmetic mean of class-specific accuracies and can be expressed as

3 $$\overline{\mu } = \frac{1}{C}\mathop \sum \limits_{i = 1}^{C} \mu_{i}$$

where $\mu_{i}$ is the class-specific accuracy, and C is the number of classes.

### Gradient-weighted class activation mapping

**Figure 7:**
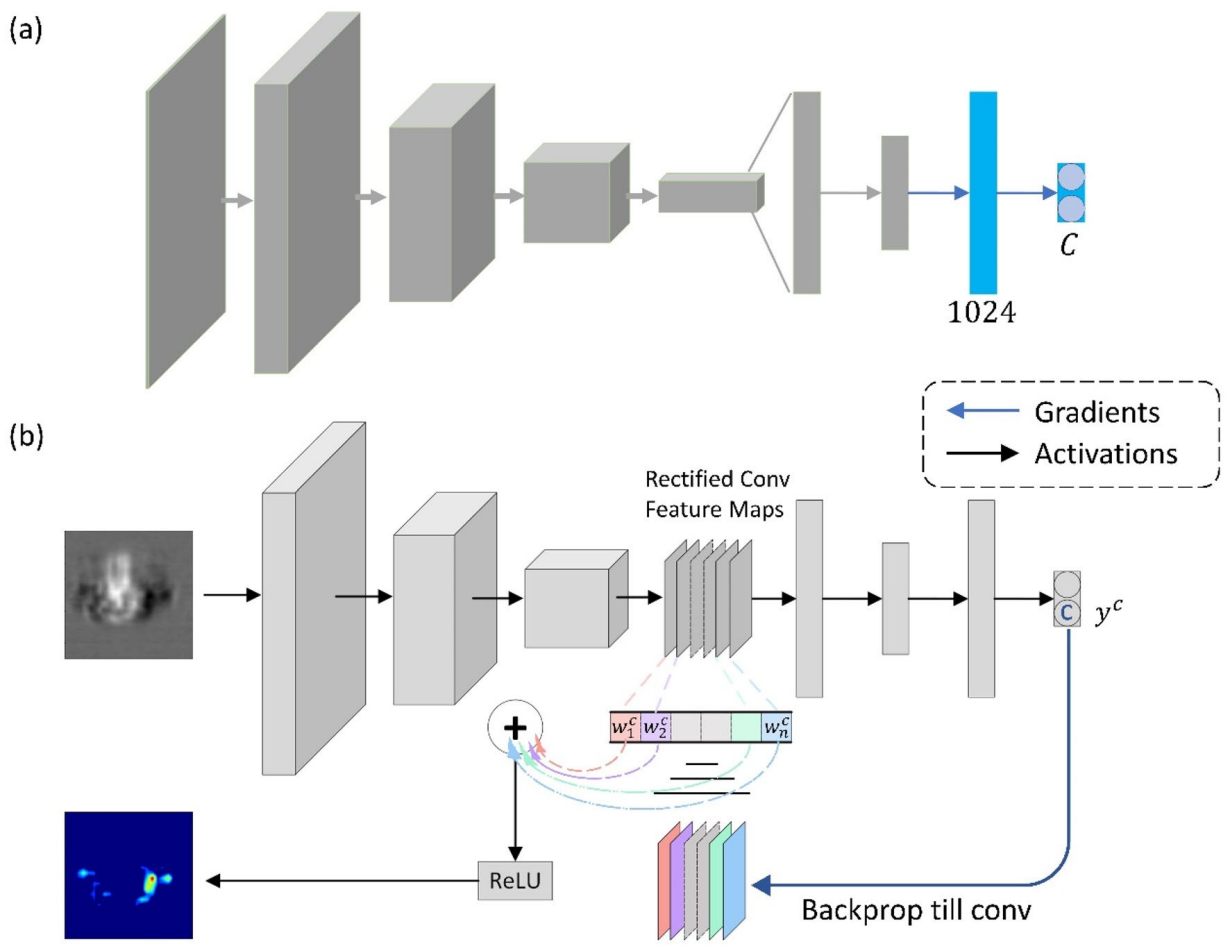
(**a**) Model architecture of the CNN classifier. The blue part denotes the fine-tuning part. (**b**) Grad-CAM method overview. The blue arrow denotes the Gradients flow. The black arrow denotes the Activations flow.

Grad-CAM is a generalization of CAM which allows us to generate visual explanations of CNN-based models^18^. To form a direct correlation between the inputs to the predictions, a CNN model containing the encoder part of the DCAE followed by two fully connected layers is trained to predict the clustering results. During the training process, the weights of the encoder part are directly transferred from the DCAE and fixed and only the last two fully connected layers are fine-tuned. Figure 7a shows the model architecture of the CNN model. To demonstrate our idea, we focus on the 2D DCAE model for the protein translocation experiment. But the method can be easily transferred to the 3D DCAE model or fused model.

In this study, we focus on the visualization of the last convolutional layer of the encoder. Figure 7b shows the workflow of how to automatically generate the Grad-CAM image. To obtain the class-discriminative localization map for any class c, the gradient score, ${w}_{k}^{c}$ for class c with respect to the k-th feature map activation, ${A}^{k}$ of a convolutional layer are first computed through gradient backpropagation^18^. The gradients are global-average-pooled and can be expressed as

4 $$w_{k}^{c} = \frac{1}{Z}\mathop \sum \limits_{i = 1}^{W} \mathop \sum \limits_{j = 1}^{H} \frac{{\partial y^{c} }}{{\partial A_{ij}^{k} }}$$

where $y^{c}$ denotes the score of class c before the SoftMax operation. The size of feature map activations $A^{k}$ are $W \times H$ and Z is the normalization factor where Z = W*H.

Then a weighted combination of forward activation maps followed by a ReLU operation is used to compute the Grad-CAM, $I_{Grad - CAM}^{c}$, which can be expressed as

5 $$I_{Grad - CAM}^{c} = ReLU\left( {\mathop \sum \limits_{k} w_{k}^{c} A_{k} } \right)$$

All deep learning models are implemented in the PyTorch framework and trained on an 8-core machine with Intel^®^ core i7-11700 K processor and NVIDIA GeForce RTX 3080 Ti with 12 GB of VRAM.

### Supplementary Information

Supplementary Information.

## Data Availability

The PyTorch Codes that support this paper have been uploaded to a public GitHub repository, Interpretable_UnsupervisedLearning.
