# Supplementary material for: Interpretable unsupervised learning enables accurate clustering with high-throughput imaging flow cytometry
Source: Sci Rep. 2023 Nov 23;13:20533. doi: 10.1038/s41598-023-46782-w (PMC10667244; doi:10.1038/s41598-023-46782-w)
Supplement: Supplementary file 1 — Supplementary Information. [file 41598_2023_46782_MOESM1_ESM.pdf]

Supporting Information for

Interpretable Unsupervised Learning Enables Accurate Clustering with High-Throughput Imaging Flow Cytometry.

Zunming Zhang[1], Xinyu Chen[1], Rui Tang[2], Yuxuan Zhu[1], Han Guo[1], Yunjia Qu[3], Pengtao Xie[1], Ian Y. Lian[4], Yingxiao Wang[3], Yu-Hwa Lo[1, *]

[1] Department of Electrical and Computer Engineering, University of California, San Diego, La Jolla, California 92093, USA; [2] NanoCellect Biomedical, Inc., San Diego, California 92121, USA; [3] Department of Bioengineering, Institute of Engineering in Medicine, University of California, San Diego, 9500 Gilman Drive, La Jolla, CA, 92093-0435, USA; [4] Department of Biology, Lamar University, Beaumont, Texas 77710, USA

[*] Author to whom correspondence should be addressed.

Email: ylo@ucsd.edu

Supporting Information Text

Mathematical Definition of Evaluation Metrics

To evaluate the clustering performance, The balanced classification precision and recall for two-class clustering experiment are defined as

$$pression = \frac{true\ positive}{true\ positive + false\ positive}$$

$$recall = \frac{true\ positive}{true\ positive + false\ negative}$$

The F_1 score can be calculated as the harmonic mean of the precision and the recall

$$F_1 = 2 \times \frac{precision\ \times recall}{precision + recall}$$

Cell Culturing and Sample Preparation Protocols

Preparation of normal white blood cells from lysing the red blood cells in the whole blood

Whole blood from healthy donors was ordered from San Diego Blood Bank (SDBB) for research purposes. White blood cells are obtained from whole blood after lysing the red blood cells (RBCs). The following steps were performed to lyse the red blood cells and to collect the white blood cells from whole blood:

1. Add 1ml of whole blood to a 15ml conical tube.
2. Add 10ml of 1X Lysis Buffer (Invitrogen, 00-4333-57) to the tube.
3. Incubate for 12mins at room temperature.
4. Spin at 400g for 6 mins.
5. Pipette out supernatant, making sure to leave no red layer in the tube.
6. Add 5ml of 1X Lysis buffer and wash at 400g.
7. Pipette out supernatant, making sure to leave no red layer in the tube.
8. Dilute sample with PBS to desired concentration. The sample should contain all WBCs and is ready for the experiment.

White blood cells staining

Antibodies, BB515 Mouse Anti-Human CD66b (Ex/Em 490/515nm, Clone G10F5, Cat. 564679, BD Biosciences) and PE anti-human CD14 Antibody (Ex/Em 496/578nm, Clone 63D3, Cat. 367103, BioLegend Biomedical Inc), were used in the white blood cell staining. CD14 is expressed at high levels on monocytes and CD66b is a glycosylphosphatidylinositol (GPI) linked protein with a molecular weight of 100 kDa expressed on granulocytes.

Staining Protocol is illustrated as follows:

1. After harvesting, cells are resuspended in 1X PBS with a concentration of approximately 3000 cells/uL.
2. Split the sample solution into Eppendorf tubes with a total volume of ~300uL. Cell concentration can be adjusted as needed in this step.
3. Add 1mL staining solution to each sample.
4. Spin down at 1500rpm for 5 min.
5. Remove the supernatant carefully without disturbing the pellet.
6. Resuspend the pellet into 85uL staining solution, pipette up and down to loosen the pellet, the recommended cell concentration for staining is approximately 2000 cells/uL.
7. Add 5uL of each antibody (cell surface marker), flick the tube to mix the solution.
8. Incubate in 4 degree C for 20mins.
9. Add 1mL 1X PBS to wash the solution.
10. Centrifuge at 1500rpm for 5mins
11. Remove the supernatant, repeat Step 9 and Step 10.
12. Remove the supernatant, resuspend in 1X PBS to desired concentration before imaging experiment.

Acute myeloid leukemia cells culturing and CellTrace CFSE Staining

The acute myeloid leukemia (AML) cells (SKNO-1) were thawed and cultured with culture media (RPMI-1640, 10% Fetal Bovine Serum, 1% Penicillin Streptomycin) in a 10 cm petri dish. SKNO-1 cells were harvested when it reached 90% confluency and resuspended to a concentration of ~1x106 cells/mL in 1X PBS. The CFSE Cell Proliferation Kit (Ex/Em 492/517nm, Cat. 34554, Thermo Fisher) was added to the cell suspension at a working concentration of 20μM. The staining process was quenched by adding fresh culture medium in the cell suspension after incubating the cells at 37℃ for 30 minutes. The cells were washed by 1X PBS and fixed by 4% paraformaldehyde solution. The fixed cells were washed and resuspended in 1X PBS before imaging.

Translocation of glucocorticoid receptor (GR) from cytosol to nucleus with dexamethasone treatment

1. After bacterial culture for 18 hours, we use the plasmid miniprep kit (D4036, Zyppy) to get pEGFP-GR (Plasmid #47504, addgene).
2. Prepare two dishes of HEK-293T cells and culture cells to ~70% confluency.
3. Use the Lipofectamine 3000 transfection kit (L3000-001, Invitrogene) to transfect cells with pEGFP-GR DNA.
4. Continue culturing for 18-24 hours and check transfection efficiency.
5. When around 40% of cells express green fluorescence protein that is bright enough, take one dish of cells for drug treatment. Dilute the stock Dexamethasone (tlrl-dex, invitrogene) with culturing media to 1μM. Keep culturing for 1 hour. Check under the microscope to make sure GFP migrates from cytoplasm to nucleus.
6. Fix both the drug-treated and untreated cells using 4% paraformaldehyde.

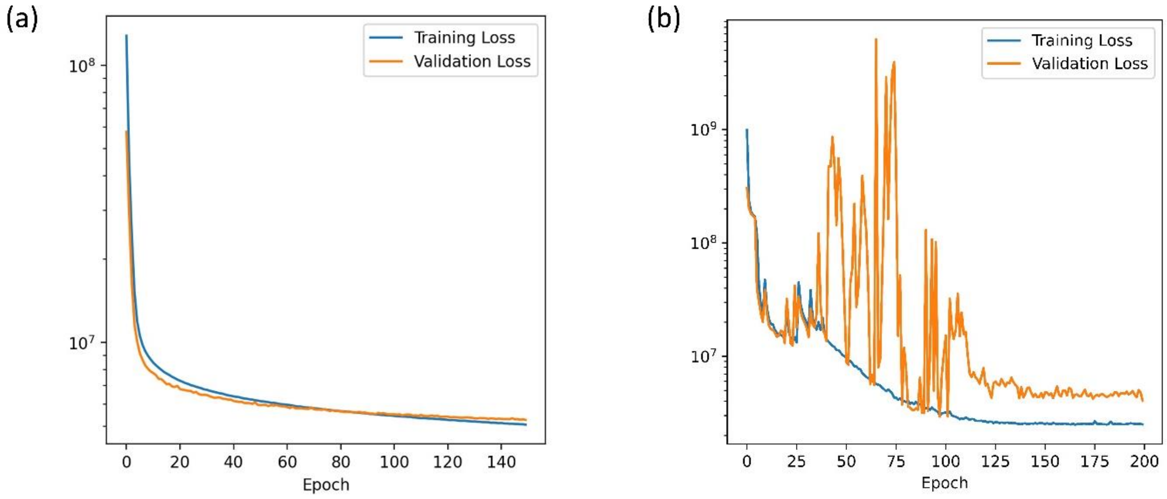

Fig. S1.

Learning curves for (a) white blood cell clustering experiment and blood cancer detection experiment; (b) protein translocation experiment.

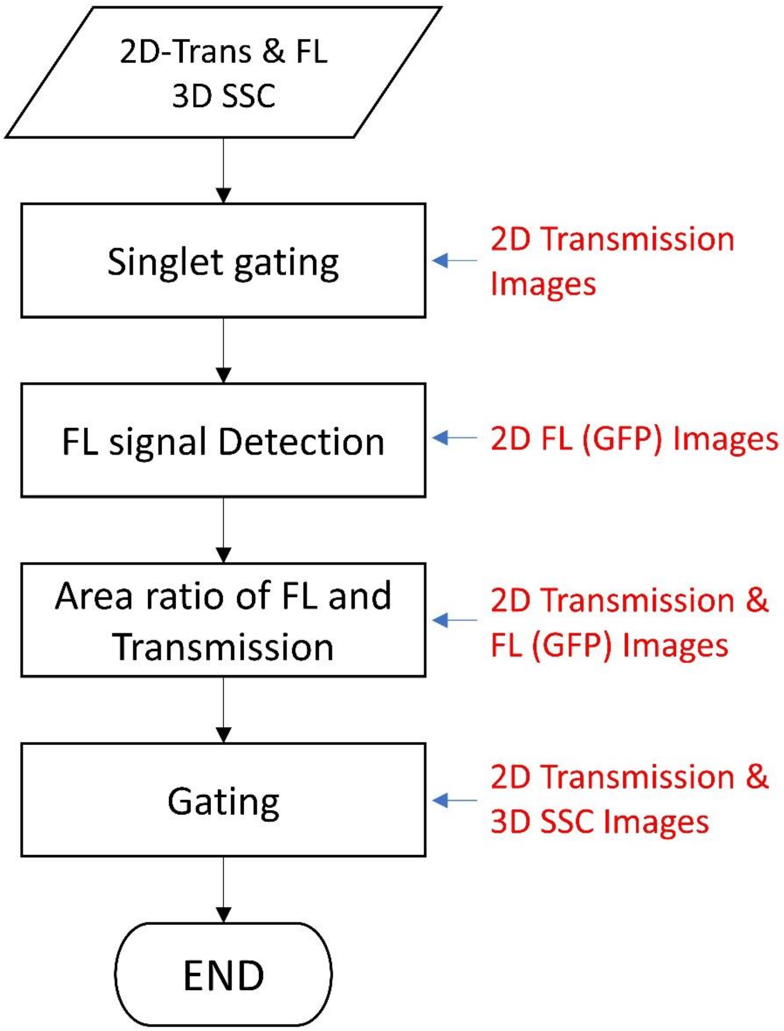

Fig. S2.
Data-processing pipeline to obtain the ground truth labels of protein translocation experiment.

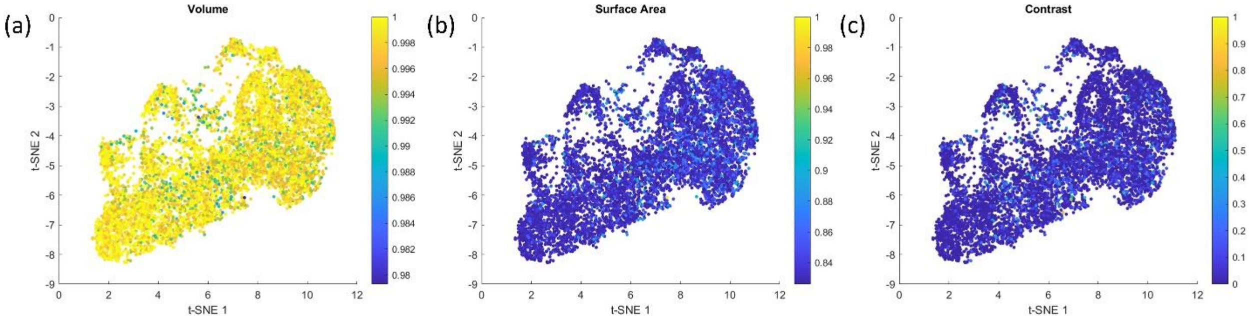

Fig. S3.

t-SNE plots for the protein translocation experiment using some cell image features such as volume, surface area, and contrast. The results show that none of these human recognizable features can distinguish protein translocated cells from non-translocated cells. (a) Volume, (b) Surface Area, (c) Contrast

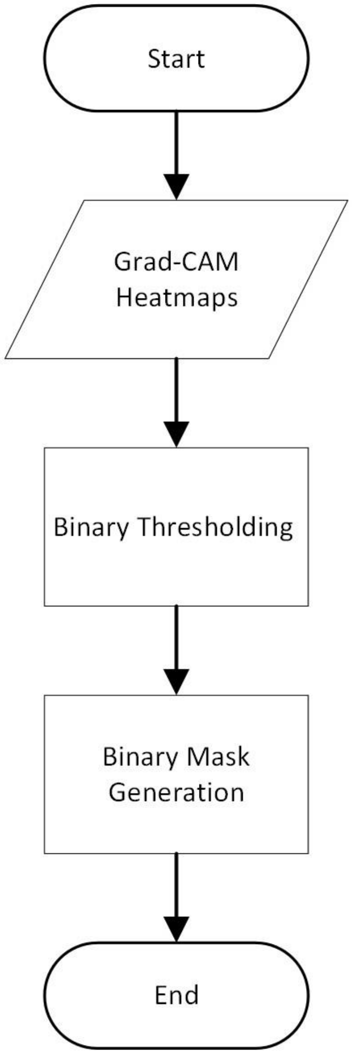

Fig. S4.
Binary Mask generation signal processing flow based on generated Grad-CAM Heatmaps. Threshold = 0.15*(max intensity).

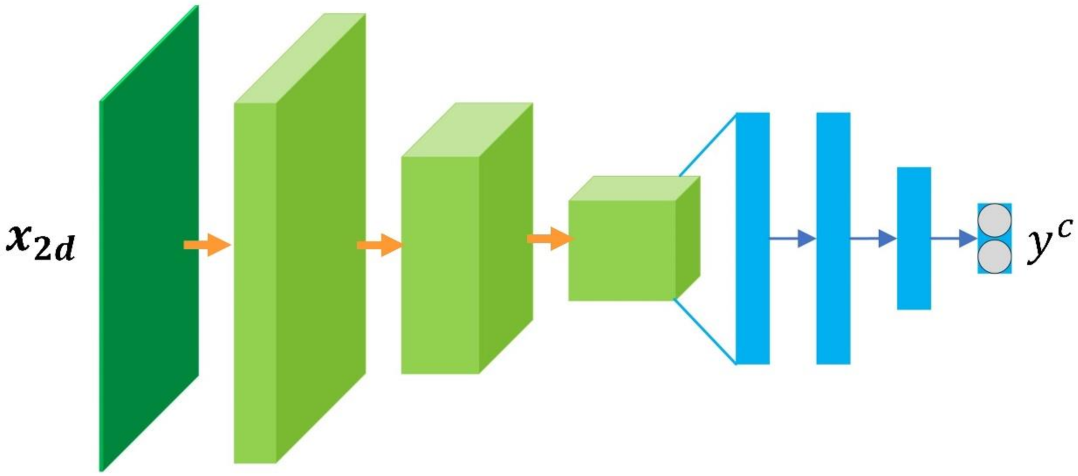

Fig. S5.

Grad-CAM generated masks evaluation CNN model architecture. The filter sizes are [1, 32, 64, 128] and the kernel size is 3X3 for each convolutional block. The fully connected layer sizes are [2014, 512, 2].

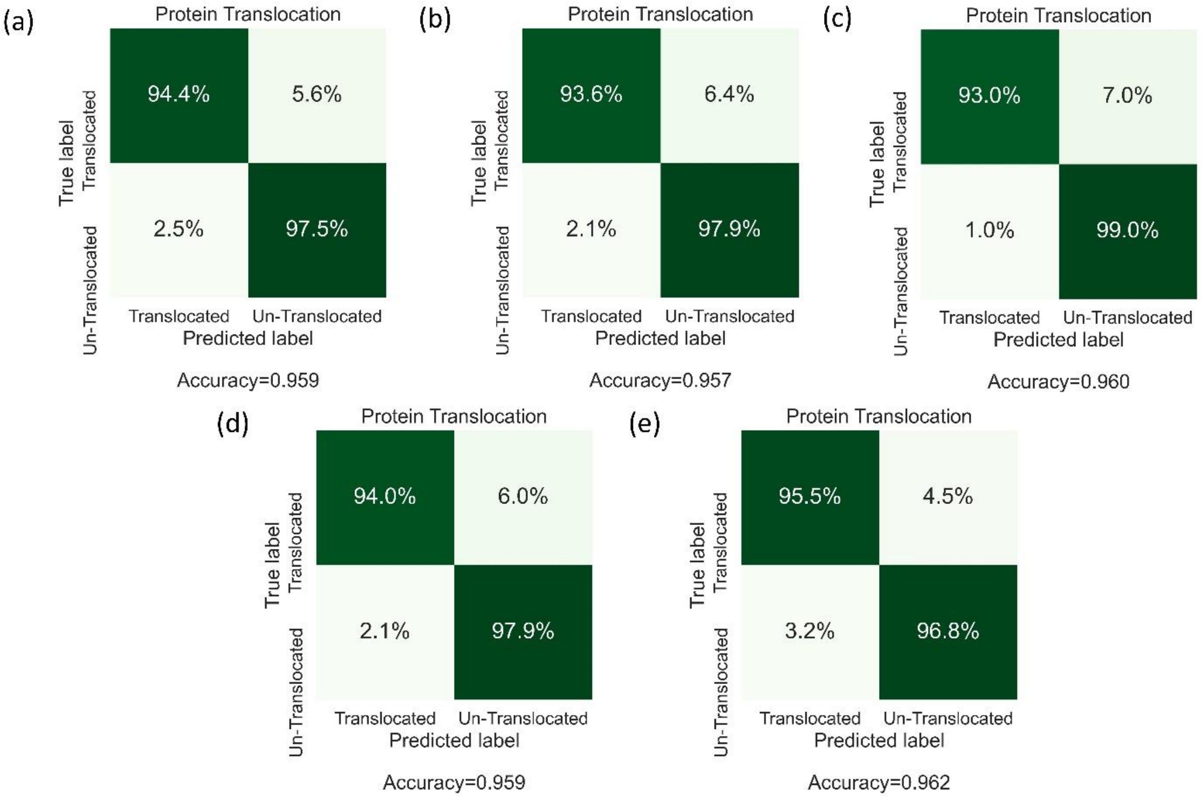

Fig. S6.

Confusion matrixes of 5-fold cross-validation results for the Grad-CAM generated mask evaluation CNN model. (a)-(e): Fold 1-5. The high accuracy (96%) shows that the Grad-CAM-generated masks are cluster-specific.

Table S1.

Full Evaluation Matrices of 5-fold cross-validation results.

Fold/Matrices	Precision macro	Precision micro	Precision weighted	Recall macro	Recall micro	Recall weighted	F1 macro	F1 micro	F1 weighted	AUC
**Fold 1**	0.962	0.962	0.962	0.960	0.962	0.962	0.961	0.962	0.962	0.985
**Fold 2**	0.962	0.961	0.961	0.958	0.961	0.961	0.960	0.961	0.961	0.988
**Fold 3**	0.968	0.965	0.966	0.960	0.965	0.965	0.963	0.965	0.965	0.990
**Fold 4**	0.964	0.963	0.963	0.960	0.963	0.963	0.961	0.963	0.963	0.988
**Fold 5**	0.962	0.963	0.963	0.962	0.963	0.963	0.962	0.963	0.963	0.987

Table S2.

Antibodies cocktail combinations for human white blood cell clustering experiment.

	**Ground Truth Labelling 1**	**Ground Truth Labelling 2**	**Unlabeled**
**Cell types**	Monocytes (PE anti-human CD14 Antibody)	Granulocytes (BB515 Mouse Anti-Human CD66b)	Lymphocytes
